# Functional Characterization of Variations on Regulatory Motifs

**DOI:** 10.1371/journal.pgen.1000018

**Published:** 2008-03-07

**Authors:** Lapidot Michal, Orna Mizrahi-Man, Yitzhak Pilpel

**Affiliations:** 1 Molecular Genetics Department, Weizmann Institute of Science, Rehovot, Israel; 2 Structural Biology Department, Weizmann Institute of Science, Rehovot, Israel; North Carolina State University, United States of America

## Abstract

Transcription factors (TFs) regulate gene expression through specific interactions with short promoter elements. The same regulatory protein may recognize a variety of related sequences. Moreover, once they are detected it is hard to predict whether highly similar sequence motifs will be recognized by the same TF and regulate similar gene expression patterns, or serve as binding sites for distinct regulatory factors. We developed computational measures to assess the functional implications of variations on regulatory motifs and to compare the functions of related sites. We have developed computational means for estimating the functional outcome of substituting a single position within a binding site and applied them to a collection of putative regulatory motifs. We predict the effects of nucleotide variations within motifs on gene expression patterns. In cases where such predictions could be compared to suitable published experimental evidence, we found very good agreement. We further accumulated statistics from multiple substitutions across various binding sites in an attempt to deduce general properties that characterize nucleotide substitutions that are more likely to alter expression. We found that substitutions involving Adenine are more likely to retain the expression pattern and that substitutions involving Guanine are more likely to alter expression compared to the rest of the substitutions. Our results should facilitate the prediction of the expression outcomes of binding site variations. One typical important implication is expected to be the ability to predict the phenotypic effect of variation in regulatory motifs in promoters.

## Author Summary

A prime mode of control of transcription is the binding of transcription factors to promoter elements. These elements are often imprecise – often more than one, yet typically not all, of the nucleotides may be tolerated. In another field of protein structures, only some of the amino acid substitutions are tolerated. Are there parallels to this situation in transcription motifs? For instance, could substitutions between Adenine and Guanine have relatively little effect on transcription? An experimental approach here is daunting, requiring a library of reporter genes, each under the regulation of a different motif version. Yet genomes contain genes with natural variations on motifs, while micro-array data provide the expression pattern of those genes. We inspected the *S. cerevisiae* genome to derive nucleotide substitution severity “rules”. We compared the expression of genes containing motifs to the expression of genes that contain motif variations, and assessed how drastically each substitution affected expression. The power of the approach comes from the statistics – we gathered data for thousands of genes and motifs in dozens of growth conditions. We found that not all nucleotide substitutions are equal: e.g. substituting Adenines is more likely to be benign, compared to Guanines. It is possible that the different chemical nature of nucleotides could explain these findings. One future implication of this type of work is that it may aid in predicting which human mutations in promoter elements are more likely to cause a disease by affecting transcription.

## Introduction

The regulation of gene expression is mediated mainly through specific interactions of TF proteins with DNA promoter elements. TF binding sites (TFBS) are short (typically of length 6–20 bases) and imprecise; unlike restriction enzymes which recognize unique nucleotide sequences, a single TF protein may interact with a range of related sequences. For most TFs, there appears to be no distinct sequence of nucleotide bases that is shared by all recognized binding sites. However, there are typically clear biases in the distribution of bases that occur at each binding site position. These biases are commonly represented by position weight matrices (PWMs), whose components give the probabilities of finding each nucleotide at each binding site position [1].

Given the degenerate nature of genuine binding sites, highly similar sites within the same genome may be recognized by the same TF or by distinct TFs. This is also true for the genomes of related species, where slight changes in binding site sequence, occurring throughout evolution, may in some cases maintain the specificity of the site and in others lead to its loss or to the creation of a site targeted by a different TF [2],[3]. The desire to distinguish between ‘neutral’ binding site variations, which do not change the recognition range of the site, and ‘functional’ variations, which may affect gene expression by altering protein-DNA interactions, lays at the heart of this work. Such a distinction may have several implications. Firstly, it should greatly improve the performance of scanning algorithms, which search promoter sequences for matches to predefined PWMs. These algorithms typically regard all mismatches between a promoter sequence and a given PWM's preferences as equal (c.f. ScanACE [4], Match™ [5], MAST [6]). More reliable predictions may be obtained if such mismatches are differentially weighed based on their expected effects on expression. Identification of genuine sites is also crucial when comparing the promoters of orthologous genes - some across-species variations may change the functionality of a motif in some of the organisms. Another intriguing application is the detection of regulatory site variations, which have the potential to reduce fitness, and cause diseases, through altering gene expression. Disease-causing binding site variations are known to occur [7],[8], however so far no attempts have been made for their prediction on a genome wide scale. Most efforts to distinguish disease-causing variations from neutral ones have focused on coding single nucleotide polymorphisms (SNPs) [9]–[15]. Estimates show that the human population contains thousands of *cis*-regulatory variations [16]. Such high numbers justify a dedicated effort for the development of computational means for predicting deleterious regulatory variations.

The present work lays the foundations for the development of such methods, experimented here in yeast, introducing measures for quantifying the effects of binding site variations on gene expression. We have first constructed a putative binding site motif collection using our previously introduced expression coherence (EC) score, which quantifies a motif's regulatory effect by measuring the extent to which genes that contain it display similar expression at a given biological condition [17]–[19]. We next exploit this quantitative measures of a motif's regulatory effect, in order to systematically compare the expression patterns of genes containing binding sites differing by a single nucleotide position.

We accumulate statistics for many substitutions across multiple putative binding sites, and observe that not all nucleotide substitutions are similar in severity: We found that substitutions of the type A->N, and substitutions of the type N->A (where N is any nucleotide but A) tend to be “benign”, i.e. they relatively rarely change the expression patterns of the regulated genes. On the other hand, substitutions of the type G->N, and N->G are more likely than other substitutions to lead to binding site loss.

## Results

### Predicting the outcome of a binding site substitution – the case of Ndt80

**Figure 1:**
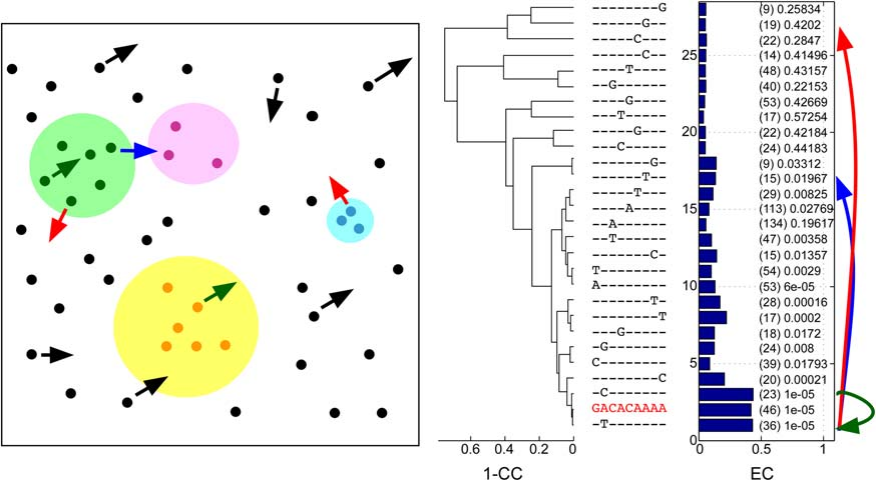
Possible outcomes of binding site substitutions. Left panel: points represent promoter elements, discs represent TF recognition ranges. Points included within a disc represent promoter elements that are bound by the corresponding TF. Arrows illustrate the result of single nucleotide substitutions within a promoter element. Three scenarios are illustrated: binding site loss (red arrows), reduced affinity to the same TF (green arrows), or binding site switching – creation of a binding site with as higher affinity to a different TF (blue arrows). Right panel: Our motif landscape analysis tool [17] is used to detect these three scenarios. This display captures the effects of motif single nucleotide substitutions on the expression profiles of the downstream genes. The analyzed motif is the yeast Ndt80 sporulation factor (consensus marked in red). The dendrogram on the left part of the display shows the similarity in mean expression profiles between gene sets bearing variations of the motif in their promoters. The right side of the display shows the similarity within sets of genes that contain the same motif variation in their promoters, as measured by the EC score. Gene set sizes appear in parentheses, and p-values corresponding to the EC scores follow. The middle section displays the sequence of the motif variation examined in the corresponding row (with a ‘-’ indicating same nucleotide as the wild type motif). A substitution that is in the recognition range of the same TF is expected to maintain a high EC score and a similar expression profile (green arrow). A substitution that causes binding site loss is expected to be recognized by both loss of coherence and a change in the mean expression profile (red arrow). A substitution that creates a motif that is in the recognition range of a different TF is expected to maintain high expression coherence, while altering the mean expression profile (blue arrow).

A single base mutation in the binding site of a TF can result in one of three scenarios: (i) Binding site conservation - the mutant is also recognized by the same TF and the substitution from wild-type to mutant is thus expected to have a very mild effect (Figure 1, green arrows). (ii) Binding site switching - the mutant is no longer recognized by the original TF, but is recognized by an alternative TF (Figure 1, blue arrows). (iii) Binding site loss: the mutant binding site is no longer recognized by any TF (Figure 1, red arrows). The primary goal of this work was to computationally distinguish these three scenarios.

As a first step towards establishing a general scheme for the assessment of the effect of single-base substitutions on the binding sites of transcription factors, we examined computationally the effects of such substitutions on the consensus site of the yeast sporulation factor Ndt80, the primary transcriptional activator of middle sporulation genes. Using our motif landscape analysis tool [17] we analyzed the effects of all single-base substitutions on Ndt80 binding (Figure 1, right panel). For each ‘mutant’ motif, this tool answers two questions: can it potentially constitute a binding site for a TF? and more specifically, is it likely to bind the same TF as the ‘wild-type’ motif? To answer the first question, the tool employs the previously described Expression Coherence (EC) score [17]–[19], which assesses the effect of a promoter sequence motif on the corresponding genes' expression profiles. The EC score measures the extent to which a set of genes (in this case the set is defined by a common sequence motif in the genes' promoters) displays similar expression profiles at a given biological condition. The statistical significance (p-value) of an EC score is the estimated probability of obtaining the observed or higher EC score by chance and is dependent on the size of the set of genes considered [17]. The answer to the second question is obtained by computing the Pearson correlation distance between the mean expression profile, under a relevant condition, of the set of genes whose promoters contain the ‘wild-type’ motif and that of the set of genes whose promoters contain the ‘mutant’ motif.

**Figure 2:**
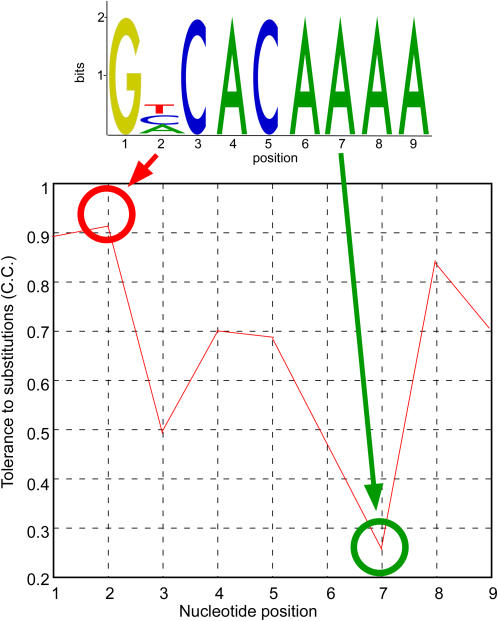
Tolerance of different Ndt80 positions to substitution. The averaged tolerance to substitution for each nucleotide position within the Ndt80 motif was defined as the averaged correlation coefficient between the mean expression profile of the genes that have a perfect match to the consensus motif and the mean expression profile of the genes that have each of the three possible substitutions relative to the consensus in that position. The second motif position appears the most tolerant to substitutions. This observation is supported by the recently published structural data of Ndt80 bound to DNA [21]. The second motif position does not form a contact with the protein.

Using the *S. cerevisiae* sporulation expression data [20], our landscape analysis predicted that two out of the three possible substitutions in the second position will have only a minor effect on expression whereas an A->G substitution at the same position will have a harsher effect (see Figure 1 legend for details). When averaging over all possible single nucleotide substitutions (Figure 2), the second position seems to be the most tolerant towards substitutions (mean expression distance, i.e. 1-Pearson correlation coefficient, is 0.0358), and the seventh position – the most sensitive (mean expression distance 0.7715). One possible reason for such a marked difference between the tolerance of different positions within the same motif to substitutions may be that the binding transcription factor forms different contacts with the DNA at each of the positions. Particularly, we may expect the positions that form tight contact to be less permissive to substitutions. Indeed, our results are in good agreement with the structural data of Ndt80 bound to its DNA target [21]: the second ‘permissive’ motif position is the only position which does not form a direct contact with the protein. But do these differences affect TF function? Reassuringly, these results are also supported by published *in vivo* reporter expression experiments and *in vitro* binding assays of Ndt80 mutants [22]. This experiment represents the ‘wet’ analog to our computational experiment – each of the nucleotide positions in the Ndt80 consensus site was replaced with all possible three alternatives. These results too showed that the second position is most permissive to substitutions, and that, as predicted by us, G is the only nucleotide that when placed at this position weakens binding affinity and reduces expression level of the reporter gene [22]. This implies that the computational measures used here can complement and predict the outcome of ‘wet’ mutation experiments.

The use of Ndt80 as a test case also provided us with the opportunity to examine whether we can computationally distinguish between binding site switching and binding site loss. Ndt80 recognizes variations of a site termed middle sporulation element (MSE), whose consensus sequence is GNCRCAAW. Interestingly, variations of the MSE are also recognized by Sum1, a transcriptional repressor of middle sporulation genes during mitosis and early sporulation. Through a combination of *in vivo* reporter expression assays and *in vitro* binding assays of Ndt80 and Sum1 mutants, Pierce et al. defined the specific binding preferences of these two TFs [22]. They found that while positions 3–5 of the MSE are important for binding of both Ndt80 and Sum1, there is a difference in binding preferences at positions 6–7. For these positions, Ndt80 requires strictly an A, whereas Sum1 binds equally to an A and to a T. Indeed, our landscape analysis (Figure 1, right panel) shows that mutating position 6 from A to T results in a change in expression profile, yet coherence remains significant (p-value 0.0083). This may be explained by the binding site switching from Ndt80 to Sum1. Transitions of the same position into C or G result in binding site loss (p-values of the EC score of the substituted motifs are 0.4 and 0.3, respectively). The same applies for position 7, in which transition from A to T maintains a relatively significant EC score (p-value 0.019), yet the expression profile is changed relative to the genes containing the consensus motif. On the other hand, substitutions at this position to both C and G lead to complete loss of coherence (EC p-values are 0.4 for both variants). This position also scored as the most sensitive to mutations – any change will abolish the Ndt80 site, either by switching or complete loss.

### Compiling a comprehensive transcription regulatory motifs collection in yeast

Encouraged by our ability to predict the effects of binding site substitutions within a single motif, we attempted to generalize these predictions in order to define universal properties of substitutions that alter gene expression. Towards this end we compiled a dataset of motifs that are likely to participate in the regulation of gene expression. This study was conducted in the *S. cerevisiae* genome, for which vast TFBS knowledge is available. However, in order to both broaden this knowledge and form a quantifiable connection between binding site sequence and the expression profiles of the regulated genes, we compiled a new comprehensive motif dataset. This dataset is unbiased by prior knowledge and is based on the premise that any nucleotide sequence that resides in the promoter of a gene may contribute to the regulation of the gene's expression. A further advantage of our dataset is that it is not limited to TF binding sites – motifs found by our methodology are relevant to transcription, but not all are necessarily TF binding sites. Some, for example, may be involved in DNA bending.

We constructed our motif dataset by integrating whole genome promoter sequences of *S. cerevisiae* with expression patterns of the corresponding genes in 40 natural and perturbed biological conditions including cell cycle, sporulation and various stress responses. Each biological condition was represented by a time series of microarrays (see methods). To obtain the most comprehensive dataset, we systematically scanned all k-mers (k ranges from 7 to 11) that appear in *S. cerevisiae* promoters. For each k-mer and each of the 40 biological conditions, we computed the EC score of the set of genes that contain it in their promoters. A p-value was assigned to each EC score and a false discovery rate (FDR) [23] of 0.1 was applied to correct for multiple hypotheses (see methods). The EC score was used not only to assess the biological significance of the scanned k-mers, but also to assign them with likely regulatory functions, in the form of the set of biological conditions in which they operate, and the regulatory effects they exert in each condition (e.g. increased expression following stress, or peak in expression level at a particular cell cycle stage).

A total of 8,610 sequence motifs appeared significant in at least one of the examined biological conditions. These comprise the ‘core’ of our dataset (hereafter referred to as the ‘core dataset’). This dataset represents potential *cis*-regulatory elements, whereas the rest of the scanned k-mers represent a control set of presumably non-functional elements. A list of our core motif sequences, along with their EC scores and p-values in the biological condition in which each motif obtained the most significant score is provided in the supporting information (supporting Table S1).

### Validation of method used to construct motif dataset

We performed several analyses to validate the ability of our method to identify biologically significant motifs (see supporting Text S1 for complete details). First, we compared the core dataset to the well accepted reference collection of yeast TFBS published by Harbison et al. [24] (supporting Text S1 and Figure S1). Briefly, the Harbison set was obtained by experimentally determining the genomic occupancy of DNA-binding transcriptional regulators under rich medium as well as other growth conditions. Using motif-discovery algorithms the information from genome-wide location data was then combined with phylogenetically conserved sequences and prior knowledge to derive for each regulator its probable specificities [24]. Each motif in the Harbison set is represented by a positional weight matrix (PWM), which specifies for each position in the regulator binding site and each of the four possible nucleotides, the likelihood of observing the specific nucleotide at that particular position. Our dataset covers 99 out of the 102 PWMs published by Harbison et al. We also found a tendency for genes, which contain the same motif from our core dataset in their promoters, to be associated with similar functions (supporting Text S1).

We next used our core dataset to investigate characteristics that may be of relevance to motifs' biological function. For this purpose we compiled a control set of 190,211 low scoring k-mers, that were insignificant in all 40 examined biological conditions, and in addition scored especially low (p-value > 0.8, gene set size > 8) in at least one of these conditions. We considered various features that may be important for the function of a regulatory motif and for each such feature, defined a quantitative measure, and tested whether it can significantly differentiate between our highly scoring motifs and the control set.

**Figure 3:**
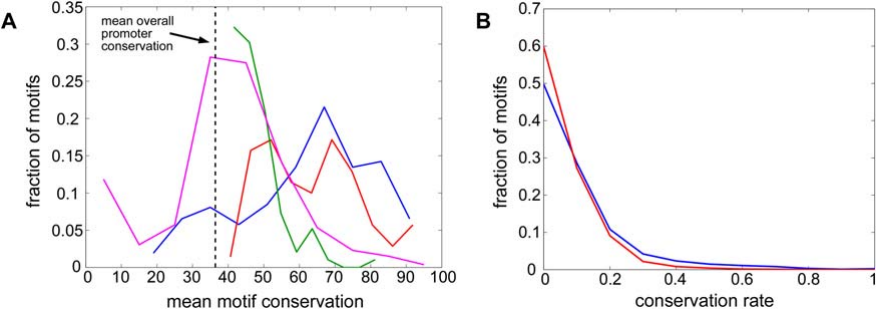
Evolutionary conservation of highly scoring k-mers. A. Evolutionary conservation in four *Saccharomyces* species [27] was calculated for the highly scoring *S. cerevisiae* cell cycle k-mers (lengths 7–11; shown in red) and compared with the conservation of two sets of motifs defined by yeast phylogenetic footprinting: Cliften et al. [27] (green) and Kellis et al. [28] (blue). A set of the randomized k-mers was used as a control (to preserve the same GC content) and is shown in magenta. For each putative motif, evolutionary conservation was calculated by finding the percentage of fully conserved (in all aligned species) positions in each motif instance, and averaging over all motif instances. The background promoter conservation was calculated in a similar manner by counting the number of fully conserved positions in each promoter and averaging over all promoters. The highly scoring k-mers are conserved comparably to the set of motifs published by Kellis et al. [28] and appear to be more conserved than the set published by Cliften et al. [27]. The randomized k-mers show an evolutionary conservation that is similar to that of the background promoters (∼36%). B. Evolutionary conservation rate in four Saccharomyces species [27] was calculated for the motifs of the core dataset (blue) and compared with the evolutionary conservation rate of a control set of randomized k-mers (to preserve GC content), shown in red. For each putative motif, evolutionary conservation rate was calculated as the percentage of motif instances that are highly conserved (i.e. at least 90% of the motif positions were identical across all four species). The evolutionary conservation rates of the core dataset motifs tend to be higher than those of the control set (Wilcoxon-Cox ranksum test; p<10^−300^ ).

Compared to the control set our significant motifs were found to have high GC content (relative to the yeast AT rich genomic background (supporting Figure S2), to have high entropy (supporting Figure S3), to appear in higher copy numbers (supporting Figure S4) and to display a preference to distinct positions relative to the transcriptional start site (TSS) in different promoters (positional bias) (supporting Figure S5). Additionally, our motifs were found to be evolutionarily conserved in the promoters of four closely related *Saccharomyces* species (Figure 3 and supporting Figure S6). Reassuringly, some of these properties are known to characterize functional binding sites (positional bias [19],[25], multiplicity of sites [17],[26], evolutionary conservation [27],[28]).

### Creation of a high-confidence subset of the core motif dataset

The analysis of evolutionary conservation revealed, that while the core dataset is significantly more conserved than a randomized motif set, there is a substantial number of motifs in the core dataset that are not significantly conserved, as manifested by an overlap in the two distributions of conservation rates (Figure 3). This implies that the core dataset likely contains many false positives. Therefore, in order to obtain a distilled signal in the analyses that follow we applied a conservation-based filter to the core dataset. Using the 95^th^ percentile of the conservation rate (see methods) of a control motif set as a threshold, we filtered out those core dataset motifs that had a conservation rate below this threshold. The new dataset, referred to as the filtered core dataset, constitutes 1,036 motifs (see supporting Table S1 for the identity of the motifs that were included in this set).

### Exploiting our dataset to predict the outcome of a binding site substitution

**Figure 4:**
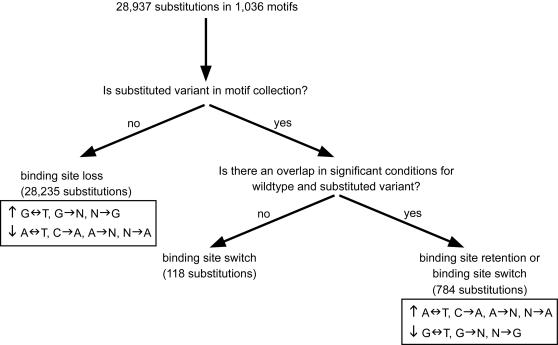
Statistics of the fates of substitutions in the filtered motif dataset. All possible single-nucleotide substitutions in the filtered motif dataset were considered. In order to obtain a non-redundant set of substitutions, if both ‘wild-type’ motif and its substituted variant were members of the filtered motif dataset then the reverse substitution was not examined. In total 28,937 substitutions were examined. Cases where the substituted variant was not a member of the filtered motif dataset were considered as binding site losses. Of the remaining 702 substitutions, 118 constitute clear cases of binding site switches, as the ‘wild-type’ and substituted variant do not share biological conditions in which both of them are presumed to be active based on their EC scores. For the remaining 584 substitutions the effect of the substitution (conservation of the binding site or binding site switch) can only be determined by the similarity in mean expression profiles, as in these cases both ‘wild-type’ and substituted variant are coherent in a shared condition. Substitution types that are over-/under-abundant in the categories of “binding site loss” or “binding site retention or binding site switch” are denoted, with upward- and downward-pointing arrows indicating over- and under-representation, respectively.

**Figure 5:**
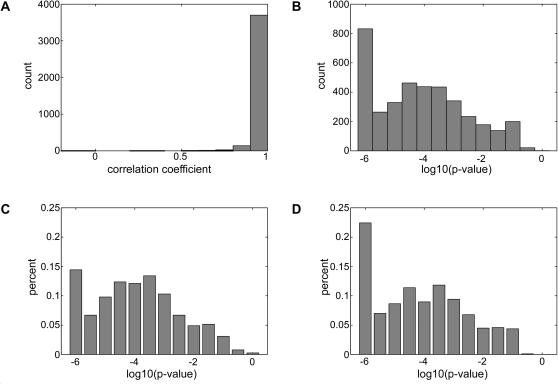
Similarity of expression patterns for overlapping conditions of single-nucleotide substitution variants. Pearson correlation coefficients (A) and corresponding p-values (B) were calculated for the mean expression profiles observed in shared conditions of pairs of motifs from the filtered core dataset that differ by a single nucleotide and share at least one condition where they both display significant expression coherence. (C) p-values for substitutions from C to G and from G to C. (D) p-values for substitutions from A to T and from T to A. Substitutions between C and G tend to produce correlations between mean expression profiles that are less significant than other substitution types. For the purpose of plotting the p-value histograms very small p-values were conservatively set to 1e-06.

We next exploited the filtered core dataset in order to study the functional outcomes of binding site variations. For each motif in this dataset we exhaustively enumerated all possible single-base substitutions and examined their effect on expression patterns, in view of the three previously described scenarios (Figure 1, right panel). The process of assessment of the effect of a substitution is best described in terms of a decision tree. Figure 4 summarizes the distribution of substitutions along the leaves of this decision tree. The first decision addresses the possibility of binding site loss. If the substitution results in a k-mer sequence that is outside of the filtered core dataset we consider the substitution to result in a binding site loss, since the genes that contain that version of the motif are not significantly coherent, whereas the genes that contain the original motif from the filtered core are coherent. Next, we attempt to discover clear cases of binding site switches. These are defined as cases where the original motif and the substituted sequence are both members of the filtered core dataset, but the sets of biological conditions in which they are inferred to exert their effect are non-overlapping. Finally, substitutions that survive the two filters can constitute either a conservation of the binding site (i.e. benign mutations) or a binding site switch, depending on their effect on the expression profile in the biological conditions in which both the motif and its substituted variant are coherent. Interestingly, examination of the distribution of correlation coefficients between the mean expression profiles of the motif and its substituted variant for those substitutions that fell into the last category revealed that these correlation coefficients tend to be high and statistically significant (Figure 5). Thus, it seems that cases similar to the one observed for Ndt80 and SUM1, in which a substitution results in a switch to a binding site that is active in the same biological condition as the original binding site, are quite rare. Table 1 describes the few cases found to constitute such a switch. Consequently, we define the substitutions that fall into the last category as benign substitutions that will have very little effect on expression. Overall from the decision tree it is apparent that the majority (∼97.5%) of the single nucleotide substitutions result in a loss of a functional binding site. It should be emphasized, though, that the our criteria for inclusion in the high confidence core dataset were very strict (both in terms of expression coherence and evolutionary conservation), and that more loose criteria could have resulted in a lower proportion of binding site loss events.

**Table 1:** Putative cases where a substitution causes a switch to a binding site that is active in the same condition as the ‘wild-type’.

motif 1	motif 2	shared biological condition	number of time points	Pearson correlation coefficient	P-value
GTGACCCG	GTGACGCG	spellman cell-cycle cdc28	17	−0.1865	0.47
CGCGTAAA	CGGGTAAA	spellman cell-cycle cdc28	17	−0.0614	0.81
CGCGACGC	GCGCCGCG	Environmental response - acid	7	0.2818	0.54
GACGCGAA	GGCGCGAA	spellman cell-cycle cdc28	17	0.3332	0.19
GCGACGCG	GCGCCGCG	Environmental response - acid	7	0.5571	0.19
ACGCGTC	GCGCGTC	Environmental response - acid	7	0.5581	0.19
CCCCTAA	CCCCTGA	Gasch environmental response - diamide	8	0.6333	0.09
GCGACGCG	GCGATGCG	Environmental response - acid	7	0.6678	0.10
ATGCGATG	TTGCGATG	Gasch environmental response - Hypo-osmotic	5	0.7401	0.15

Nine cases are described where two motifs, which are members of the filtered dataset, differ by one nucleotide from each other, and show significant coherence in a shared biological condition. The mean expression profiles of genes harboring these motifs in their promoters are different (Pearson correlation coefficient < 0.75 and p-value>0.05), implying that the substitution that would switch one motif to the other would cause a binding site switch.

### Deducing general properties of expression-altering substitutions

Using the classification of substitutions obtained for the filtered core dataset we next attempted to generalize these predictions in order to define properties of substitutions that alter gene expression. Our goal was to define substitution types that are more radical than others (in analogy to amino acid substitutions where there are conservative changes that maintain the chemical properties of the residue versus radical changes that result in a residue with different characteristics). For this purpose we used chi-square tests to compare the distribution of the different substitution types among the cases of binding site loss with that found for cases with benign effects on expression (the last category in the decision tree which corresponds to binding site conservation or switch). The results are summarized in Tables 2–4. We found that substitutions from A to T (or from T to A) are the most underrepresented among the cases of binding site loss, implying that such substitutions tend to be benign (Table 2). On the other hand, substitutions from G to T (or from T to G) seem to be very radical as they are the most overrepresented among cases of binding site loss (Table 2). In general it seems that substitutions that involve a G (either as the source nucleotide or as a target nucleotide, i.e. N->G, or G->N, where N is any nucleotide, but G) tend to lead to a binding site loss, whereas substitutions that involve an A (as a source or as a target) have a tendency to be benign (see Tables 3 and 4 for source and target statistics, respectively). Also, the identity of the source nucleotide has less of an impact on the outcome than the identity of the target nucleotide, as attested by the higher chi-square scores in Table 4 compared to Table 3. Finally, by taking into account the chi-square deviation observed for each of the twelve possible substitution types, as well as the information whether it is found more or less than expected among the cases of binding site loss, we can rank the substitution types from most benign to most severe (Table 2). These statistics also allow us to rank in terms of severity the three possible substitutions from every source nucleotide. For instance, in a position containing a C as the source, the most severe substitution would be to a G, followed by T, and then by A.

**Table 2:** Comparison of the distributions of substitution types among cases of binding site loss and those of benign effect.

source nucleotide	target nucleotide	cases of binding site loss	cases of benign effect^a^	more/less binding site loss compared to expectation	chi-square deviation	p-value
T	A	2392	80.5	**less**	20.59	**5.69e-06**
A	T	2558	80.5	**less**	15.36	**8.91e-05**
C	A	2142	67.5	**less**	12.75	**3.56e-04**
A	C	2606	67.5	less	3.69	0.0549
C	T	2147	44.5	less	2.04e-04	0.9886
G	A	2196	41	more	0.46	0.4986
T	C	2476	44.5	more	0.95	0.3305
C	G	2180	35	more	2.41	0.1208
G	C	2212	35	more	2.70	0.1005
A	G	2623	41	more	3.51	0.0609
G	T	2206	23.5	**more**	11.51	**6.93e-04**
T	G	2497	23.5	**more**	16.65	**4.49e-05**

The counts of the different substitution types are displayed for the inferred cases of binding site losses and benign effect (‘benign’ are defined as cases where both the original motif and the substituted variant share at least one condition in which both display significant EC scores). The distributions of counts differ significantly among the two types of substitution outcomes (chi-square test; chi-square deviation = 82.89; 11 degrees of freedom; p-value = 4.07e-13). For each substitution type we also tested whether it is distributed differently among the two categories of substitution outcomes, using a chi-square test with one degree of freedom (for the purpose of this test all other substitution types were collapsed into one category of substitution types). Statistically significant p-values, using a FDR [23] of 0.1, are shown in bold. The table also specifies whether the substitution type is found more/less than expected among the cases of binding site loss. Using the direction and size of the deviation from expectation the substitution types are sorted in ascending order of predicted severity.

a For cases where the substitution results in a benign outcome, i.e. both original motif and the substituted variant are within the filtered motif dataset, the identity of the original motif and the mutant was decided according to the order of traversal of the motif dataset (in order to avoid double-counting of substitutions). Therefore, in order to avoid the dependence of counts on the order of processing of motifs, counts for complementary substitution types (e.g. C->G and G->C) were averaged.

**Table 3 pgen-1000018-t003:** Comparison of the distributions of substitution types among cases of binding site loss and those of benign effect, pooling together substitution types with the same source nucleotide.

source nucleotide	cases of binding site loss	cases of benign effect^a^	binding site loss more/less than expectation	chi-square deviation	p-value
A	7787	189	less	6.5415	**0.0105**
C	6469	147	less	1.6522	0.1987
G	6614	99.5	more	13.0631	**3.01e-04**
T	7365	148.5	more	0.128	0.7205

The counts of the different substitution types, pooling together substitutions with the same source nucleotide, are displayed for the inferred cases of binding site losses and benign effect (cases where both the original motif and the substituted variant share at least one condition in which both display significant EC scores). The distributions of counts differ significantly among the two types of substitution outcomes (chi-square test; chi-square deviation = 16.1185; 3 degrees of freedom; p-value = 0.0011). For each substitution type we also tested whether it is distributed differently among the two categories of substitution outcomes, using a chi-square test with one degree of freedom (for the purpose of this test all other substitution types were collapsed into one category of substitution types). Statistically significant p-values, using a FDR [23] of 0.1, are shown in bold. The table also specifies whether the substitution type is found more/less than expected among the cases of binding site loss.

a For cases where the substitution results in a benign outcome, i.e. both original motif and the substituted variant are within the filtered motif dataset, the identity of the original motif and the mutant was decided according to the order of traversal of the motif dataset (in order to avoid double-counting of substitutions). Therefore, in order to avoid the dependence of counts on the order of processing of motifs, counts for complementary substitution types (e.g. C->G and G->C) were averaged.

**Table 4:** Comparison of the distributions of substitution types among cases of binding site loss and those of benign effect, pooling together substitution types with the same target nucleotide.

target nucleotide	cases of binding site loss	cases of benign effect^a^	binding site loss more/less than expectation	chi-square deviation	p-value
A	6730	189	less	22.8045	**1.79e-06**
C	7294	147	less	0.1309	0.7175
G	7300	99.5	more	23.307	**1.38e-06**
T	6911	148.5	more	0.28	0.5967

The counts of the different substitution types, pooling together substitutions with the same target nucleotide, are displayed for the inferred cases of binding site losses and benign effect (cases where both the original motif and the substituted variant share at least one condition in which both display significant EC scores). The distributions of counts differ significantly among the two types of substitution outcomes (chi-square test; chi-square deviation = 34.9607; 3 degrees of freedom; p-value = 1.24e-07). For each substitution type we also tested whether it is distributed differently among the two categories of substitution outcomes, using a chi-square test with one degree of freedom (for the purpose of this test all other substitution types were collapsed into one category of substitution types). Statistically significant p-values, using a FDR [23] of 0.1, are shown in bold. The table also specifies whether the substitution type is found more/less than expected among the cases of binding site loss.

a For cases where the substitution results in a benign outcome, i.e. both original motif and the substituted variant are within the filtered motif dataset, the identity of the original motif and the mutant was decided according to the order of traversal of the motif dataset (in order to avoid double-counting of substitutions). Therefore, in order to avoid the dependence of counts on the order of processing of motifs, counts for complementary substitution types (e.g. C->G and G->C) were averaged.

We further examined the cases where two motifs that differ by one nucleotide are coherent in a shared condition. In such cases the genes that contain one version of a motif and the genes that contain an alternative version of the same motif may display a similar, or dissimilar expression pattern (in the same condition where they both show high coherence). We thus examined the corresponding expression profiles in such conditions and asked if they tend to be similar or not for the different substitution types. To explore this possibility we examined separately the distributions of correlation coefficients and corresponding p-values for the different substitution types (supporting Figure S7). Since we cannot distinguish which motif corresponds to the ‘wild-type’ and which corresponds to a ‘mutant’ in this case the distributions for complementary substitution types were pooled together. Visual examination of these distributions revealed that the distribution of p-values for substitutions between C and G tends to higher (i.e. less significant) values relative to the corresponding distributions for substitutions among other pairs of nucleotides (median p-value for C⇔G substitutions is 1.08e-04, whereas the highest median for the other pairs of nucleotides is 6.60e-05). In other words, substitutions between G and C tend to generate more cases of dissimilar expression patterns compared to other substitutions. Using a rigorous comparison between the distributions corresponding to different pairs of nucleotides we found that no two distributions differ significantly from each other when correcting for multiple hypotheses (supporting Tables S2, S3, and S4). Nonetheless, the comparisons between the distribution of p-values corresponding to C⇔G substitutions to the other distributions yield much lower p-values than comparisons among the distributions of other pairs of nucleotides (p-values range from 0.0336 to 0.1606 for comparisons involving the C⇔G substitutions, whereas for other comparisons the lowest p-value is 0.5382). This may indicate that C⇔G substitutions have a greater tendency than other substitutions to weaken the affinity of the regulatory protein to the binding site.

## Discussion

We constructed an unbiased dataset of motifs relevant to transcription in the yeast genome by quantifying the effect of promoter sequence elements on the expression profiles of the corresponding genes. We validated the biological significance of our putative motifs by multiple analyses including coverage of known TF binding sites, evolutionary conservation and additional features, which are known to characterize functional binding sites. The quantitative link we formed between motif sequence and function allowed us to compare the effects on gene expression of binding sites differing by a single nucleotide position. Such comparisons were then used to infer what would be the severity of substituting one binding site into the other. We applied our tools to the yeast genome and were able to produce reliable predictions about the outcome of single nucleotide substitutions in a single binding site, of the transcription factor Ndt80, that has been extensively characterized experimentally. Applying the same rationale to a high-confidence subset of our motifs, defined by its evolutionary conservation, we were able to assess the effects of single-base substitutions on motifs from this subset and examine the prevalence of binding site losses, binding site switches and binding site retention among the substitution outcomes. We found that the overwhelming majority of single nucleotide substitutions result in binding site loss. However, the flip side of the coin is that many sequences that do not participate in transcription regulation are only a single nucleotide substitution away from becoming a functional regulatory site. The *de novo* formation of such sites might have a detrimental effect, and may explain the fact that motifs that are assumed to be non-functional differ in their positional preferences from our core dataset of motifs (see supporting Text S1 and Figure S5). It would be interesting to examine whether non-functional motifs that differ by only a single nucleotide from functional motifs are specifically avoided at promoter positions that are relevant for transcription regulation.

### Differential effects of substitution types on binding sites

By accumulating statistics for many substitutions across the motifs in our filtered motif dataset we observed that not all nucleotide substitutions are similar in severity: In the *S. cerevisiae* genome substitutions involving a G have a harsher effect on average than those involving an A. This observation may be perhaps explained by the fact that although both G and A participate in specific protein-DNA recognition through hydrogen bonds between the side chains of nucleotides and amino acids, the amino acids that preferentially bind G (Lysine, Arginine, Serine and Histidine) are much more prevalent in DNA-protein contacts than those that preferentially recognize A (Asparagine and Glutamine) [29]. Also, the observation that substitutions between A and T are under-abundant in cases of binding site loss may be explained by the fact that both these nucleotides participate in ring-stacking interactions with proline and phenylalanine in protein-DNA complexes [29]. It would be interesting to check whether the rules we have found apply to additional genomes.

An intriguing follow-up on this study would be to test additional features that may affect the sensitivity of a binding site position to substitutions, such as its evolutionary conservation and its proximity to the protein in the DNA-protein complex. Many such features may be ultimately integrated in order to form a prioritization scheme that would allow the ranking of existing genome variations by their disease-causing potential.

The approach presented here demonstrates for the first time how a huge amount of data, in this case the promoter sequences of all yeast genes as well as expression data for all genes across multiple conditions, can be harnessed and utilized for taking the first step towards assessing the effects of nucleotide substitutions on regulatory binding sites. A conceptual analogue of this endeavor for assessing the effects of amino acid substitutions on protein function could amount to mutating many proteins, say enzymes, in many different ways, and for each mutation measuring the reduction, or change, in biochemical activity and specificity. Since data for such an effort is not even close to becoming available, the methodology presented here utilizes in a unique way data that is available for its domain. While the main advantage of our methodology is the huge sample size, the disadvantage is that we are unable to control for other differences between promoters of analyzed genes (i.e. differences that are outside of the substituted position). The fact that we obtain statistically significant differences between the effects of different types of substitutions on expression likely indicates that, despite uncontrolled sources of variation, we extracted genuine signals.

An additional application of the present approach may be in algorithms that assign PWMs to promoters (e.g. PRIMA [30]) as it should provide means to differentially weigh mismatches between the PWM preferences and the promoter sequence, based on expected effect on expression. Particularly, at least in the *S. cerevisiae* genome, if the mismatch between the PWM and the sequence examined involves a G, the sequence is less likely to be a functional binding site than if the mismatch involves an A.

## Methods

### Dataset construction

Promoter sequences for 5,651 S. cerevisiae genes were taken from the Saccharomyces Genome Database (SGD) [31]. Whole-genome mRNA expression data of 40 time series experiments in S. cerevisiae, were downloaded from ExpressDB [32]. These time series represent a wide range of natural (e.g. cell cycle) and perturbed conditions. This set of conditions was utilized by us before [33] and a complete list of conditions is available at http://longitude.weizmann.ac.il/TFLocation/conditions_explist.html.

Yeast promoters were systematically scanned for all occurrences of every possible k-mer (k varies from 7–11), resulting in an index file listing for each k-mer the set of genes that contain it in their promoters, along with the positions and orientations (strand) of each occurrence. Bidirectional promoters were taken twice in different orientations and associated with the corresponding genes. Following the k-mer indexing step, EC scores (and corresponding p-values) in various experimental conditions were calculated for the sets of genes containing each of the k-mers in their promoters. The correction for multiple hypotheses was performed separately for each condition using a false discovery rate (FDR [23]) of 0.1 (allowing 10% false positives). In addition to the EC scores and corresponding p-values, each k-mer was characterized by the expression profile it dictates; this was defined, at each time point as the average expression level of all genes assigned to the k-mer. Such averaged profiles were defined for each k-mer in each of the 40 time series experiments, resulting in 40 vectors per motif.

A fundamental assumption made by our method was that a regulatory protein recognizes and binds double stranded DNA, and would therefore bind a motif instance equally whether it appears on the forward strand or the reverse strand of the promoter. Thus, in generating our motif dataset we considered a specific k-mer and its reverse complement as a single motif. As a consequence, pairs of genes with divergent promoters always ended up together in the sets of genes used to calculate the EC score of a motif. To address the question of what impact the above assumption had on the resulting dataset of motifs with statistically significant EC scores (the ‘double-stranded dataset’), we reapplied our method to the promoters of the *S. cerevisiae* this time counting forward instances and reverse instances of the same k-mer separately. We refer to the dataset of motifs with statistically significant EC scores generated in this way as the ‘single-stranded dataset’. Supporting Table S5 gives a detailed comparison of the double-stranded and single-stranded datasets. While the two datasets are of similar size, the overlap between them is quite low: 4728/8610 motifs are unique to the double-stranded dataset (both the forward and reverse instance of the motif is absent from the single-stranded dataset) and 3767/8280 motifs are completely unique to the single-stranded dataset. This indicates that the answer to the question of whether TF binding sites are directional is not a straight-forward one. The high fraction of motifs unique to the double-stranded dataset implies that in many cases both forward and reverse instances of a motif generate the same expression pattern, and may not be detectable using the single stranded methodology because of lack of statistical power due to splitting of the regulated genes into two gene sets. On the other hand, the high fraction of motifs unique to the single-stranded dataset, as well as the high number of cases where only one direction of a double-stranded motif is included in the single-stranded dataset implies that in many cases the directionality of the motif is important to the recognition by the regulatory protein. For these motifs consideration of their reverse complement in the calculation of EC scores in the double-stranded methodology adds noise that in many cases cannot be overcome by the signal. Thus, the most comprehensive approach in the future would probably be to consider the union of the results obtained using both the double-stranded and the single-stranded approaches. For simplicity, we chose to limit the current study to those motifs generated by the double-stranded methodology. Future studies may consider also those motifs generated using the single-stranded methodology.

Our method for compiling our dataset of potential regulatory motif is based on the detection of motifs whose presence in the promoters of a group of genes is associated with a similar expression pattern for the genes in question. A potential concern could be that paralogs resulting from very recent duplication events, and that have therefore not diverged sufficiently both in their promoters and expression patterns, would lead to false positives in our motif dataset. To address this concern we examined, using the data of Kafri et al. [34], the relationship between time of duplication (as assessed by Ks, the rate of synonymous substitutions within the coding sequence) and the overlap in known motif content of pairs of paralogs (supporting Figure S8). We found that time of duplication is not a good predictor for the tendency of paralogs to share regulatory motifs. Therefore, it seems unlikely that the similarity in promoter sequences of paralogous genes would drive false discovery of potential regulatory motifs.

### The Expression Coherence (EC) score

The formal definition of the EC score is the fraction of gene pairs in a given set S, for which the Euclidean distance between normalized expression profiles falls bellow a threshold D.
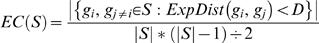



The threshold D is determined based on the distribution of pairwise distances between expression profiles of all genes in the genome (or more precisely of all genes for which expression level was monitored). The original definition of the EC score [18] used the 5th percentile as the cutoff for defining “close” expression profiles (D). This definition may create a bias towards TFs that exert a very tight regulation and miss regulatory motifs that correspond to factors exerting a more lose regulation. We therefore tested a range of EC definitions, with cutoffs corresponding to the 5th, 10th, 20th, 30th, 40th and 50th percentiles of the pair-wise distance distribution. For each definition of EC cutoff we assigned a significance p-value separately. P-values were calculated by random sampling. For each of the 40 expression time series and for each gene set size (varying from 3–50 genes), we selected 100,000 random gene sets and computed an EC score for each such set at each cutoff definition. We define the p-value of a given EC score as the fraction of random sets (of the same size and at the same condition) that scored similarly or higher (note that this sets a lower bound of 10^−5^ on the significance that can be assigned to a given EC score). Because we assume that for a given EC score, the probability to get the same score for random sets of genes drops with the set size, gene sets larger than 50 were assigned an upper bound approximated p-value, using the randomly sampled sets of size 50. See supporting Figure S9 for the distribution of set sizes used for the different k-mers.

### Evolutionary conservation

Promoter data for four closely related *Saccharomyces* species *S. cerevisiae*, *S. mikatae*, *S. kudriazevii and S. bayanus* were taken from Cliften et al. [27]. Reference lists of motifs that were defined solely based on phylogenetic footprinting were taken from both Cliften et al. [27] and Kellis et al. [28]. The motif conservation calculation was adapted from Xie et al. [35]. Motif conservation was defined as the fraction of motif positions that are identical across all 4 species. We defined the motif conservation rate separately for each motif as the ratio of conserved motif instances to total occurrences of the motif in the genome. We regarded a motif instance as conserved if it displayed at least 90% conservation. Note that since the promoter alignments do not cover whole promoters for all genes the conservation rate doesn't factor in all occurrences of the motif in the *S. cerevisiae* genome, and in particular some of the motifs did not appear in any of the alignments and their conservation rate is thus undetermined. The distribution of conservation rates obtained for our core dataset of motifs was compared to a control distribution, which was obtained by calculating the conservation rate for randomized versions of the motifs (in order to preserve GC content). We took the 95th percentile of the control set distribution as the cutoff defining high conservation. Supporting Protocol S1 displays the results obtained with different cutoffs for the definition of high conservation. It can be seen that using different cutoffs the general trends observed in Table 2 are preserved, albeit less strictly in the more permissive cutoffs.

## Supporting Information

Figure S1 Re-discovery of the Harbison motif set using our scoring method
(0.07 MB DOC)

Figure S2 Distributions of GC content for high scoring k-mers and for the Harbison motifs
(0.09 MB DOC)

Figure S3 Distributions of entropy values and number of different nucleotides within the k-mer for high scoring k-mers versus low scoring k-mers
(0.09 MB DOC)

Figure S4 Distributions of mean and maximum number of occurrences per promoter for high scoring k-mers versus low scoring k-mers
(0.07 MB DOC)

Figure S5 Positional bias of high scoring k-mers.
(0.15 MB DOC)

Figure S6 Plot of normalized EC scores versus evolutionary conservation for the highly scoring S. cerevisiae cell cycle k-mers.
(0.09 MB DOC)

Figure S7 Distribution of expression similarities for the different types of single nucleotide substitutions
(0.04 MB DOC)

Figure S8 Comparison of the age of duplication of pairs of paralogs (approximated by Ks) and their tendency to share known regulatory motifs in their promoters
(0.12 MB PDF)

Figure S9 Distribution of the set sizes of genes examined in constructing the core motif dataset that is relevant for the cell cycle
(0.03 MB DOC)

Table S1 Significantly scoring k-mers (our core set motifs). This table lists our core motif sequences, along with their EC scores and p-values in the biological condition in which each motif obtained the most significant score. For motifs that matched at least one of Harbison's PWMs with a match score higher than 99, the highest scoring match is also listed.
(1.41 MB XLS)

Table S2 Redundancy and uniqueness in the core motif dataset
(0.05 MB DOC)

Table S3 Coverage of the Harbison motif set by our core dataset
(0.04 MB DOC)

Table S4 comparison, among the different substitution types, of the distributions of p-values (corresponding to correlation coefficients) of mean expression profiles (in shared conditions) of motifs differing by a single nucleotide substitution
(0.01 MB XLS)

Table S5 Comparison of the significant motif datasets obtained using the double-stranded and single-stranded methodologies
(0.04 MB XLS)

Text S1 Validation of the method of construction of the motif dataset and the comparison of this dataset to published datasets.
(0.14 MB DOC)

Protocol S1 Robustness Analysis
(0.05 MB XLS)
