# Correction: Functional Characterization of Variations on Regulatory Motifs

**DOI:** 10.1371/annotation/99662172-8f18-4bfb-ad0f-79ed1d69fffd

**Published:** 2008-06-02

**Authors:** Michal Lapidot, Orna Mizrahi-Man, Yitzhak Pilpel

Michal Lapidot's name was incorrectly listed as Lapidot Michal. The correct citation is: Lapidot M, Mizrahi-Man O, Pilpel Y (2008) Functional Characterization of Variations on Regulatory Motifs. PLoS Genet 4(3): e1000018. doi:10.1371/journal.pgen.1000018

