# Supplementary material for: Functional Characterization of Variations on Regulatory Motifs
Source: PLoS Genet. 2008 Mar 7;4(3):e1000018. doi: 10.1371/journal.pgen.1000018 (PMC2265473; doi:10.1371/journal.pgen.1000018)
Supplement: Figure S1 — Re-discovery of the Harbison motif set using our scoring method (0.07 MB DOC) [file pgen.1000018.s001.doc]

| 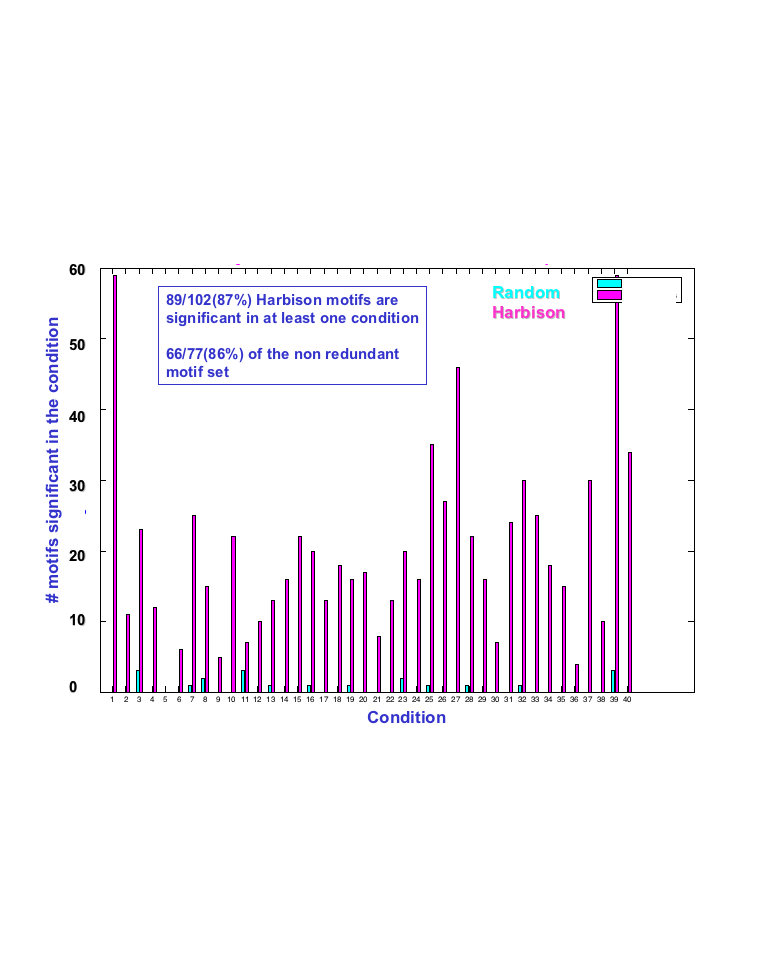 |
| --- |

## Figure s1 - Re-discovery of the Harbison motif set using our scoring method

Bar plot depicting the number of Harbison motifs (pink) which obtained significant EC scores, versus a control of random gene sets of similar sizes (cyan). Significance was tested across 40 experimental conditions (x axis). Not all conditions require the same amount of regulators, the largest number of Harbison’s TFs appear to regulate (i.e. obtain significant EC scores in the corresponding experiments) cell cycle, nitrogen depletion, oxidative stress (in response to menadione, a superoxide-generating drug), and hypo-osmotic shock.
