# Supplementary material for: Functional Characterization of Variations on Regulatory Motifs
Source: PLoS Genet. 2008 Mar 7;4(3):e1000018. doi: 10.1371/journal.pgen.1000018 (PMC2265473; doi:10.1371/journal.pgen.1000018)
Supplement: Figure S2 — Distributions of GC content for high scoring k-mers and for the Harbison motifs (0.09 MB DOC) [file pgen.1000018.s002.doc]

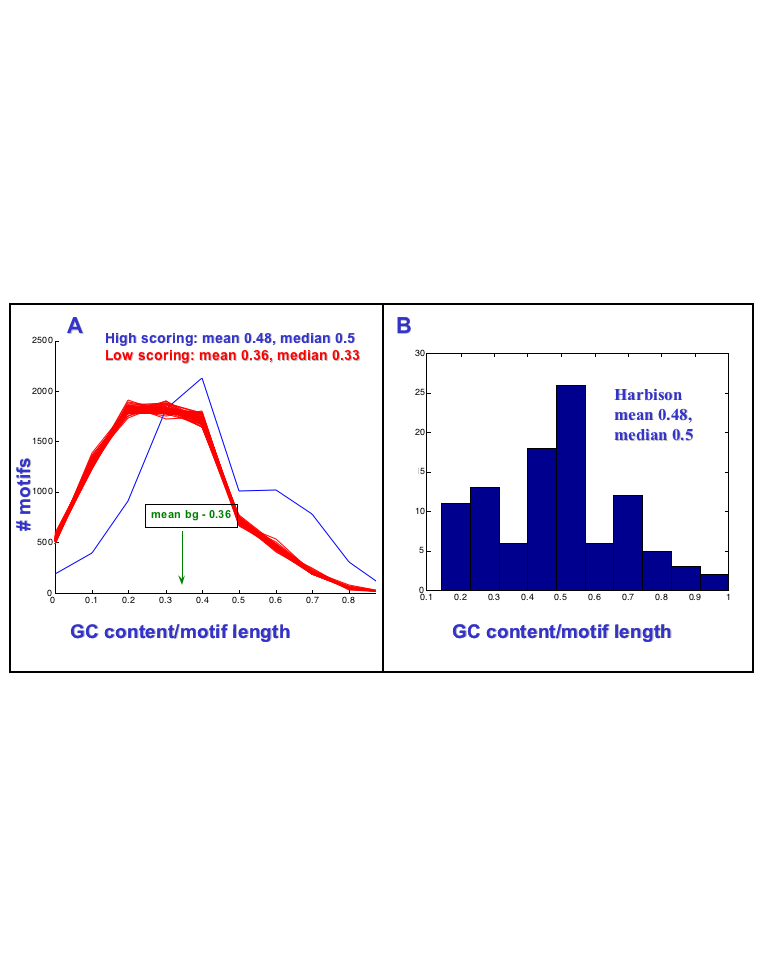


**Figure S2:** A. Distributions of GC content for high scoring k-mers and for 50 random sets of control k-mers. The distributions differ significantly (P<10^-300^). The control k-mers peak very close to the background promoter GC content (0.36), whereas the high scoring k-mers are more GC rich

B. Distribution of GC content for Harbison motifs. The normalized GC content of the high scoring k-mers is similar to that of Harbison’s set, and significantly higher than that of the control motifs
