# Supplementary material for: Functional Characterization of Variations on Regulatory Motifs
Source: PLoS Genet. 2008 Mar 7;4(3):e1000018. doi: 10.1371/journal.pgen.1000018 (PMC2265473; doi:10.1371/journal.pgen.1000018)
Supplement: Figure S3 — Distributions of entropy values and number of different nucleotides within the k-mer for high scoring k-mers versus low scoring k-mers (0.09 MB DOC) [file pgen.1000018.s003.doc]

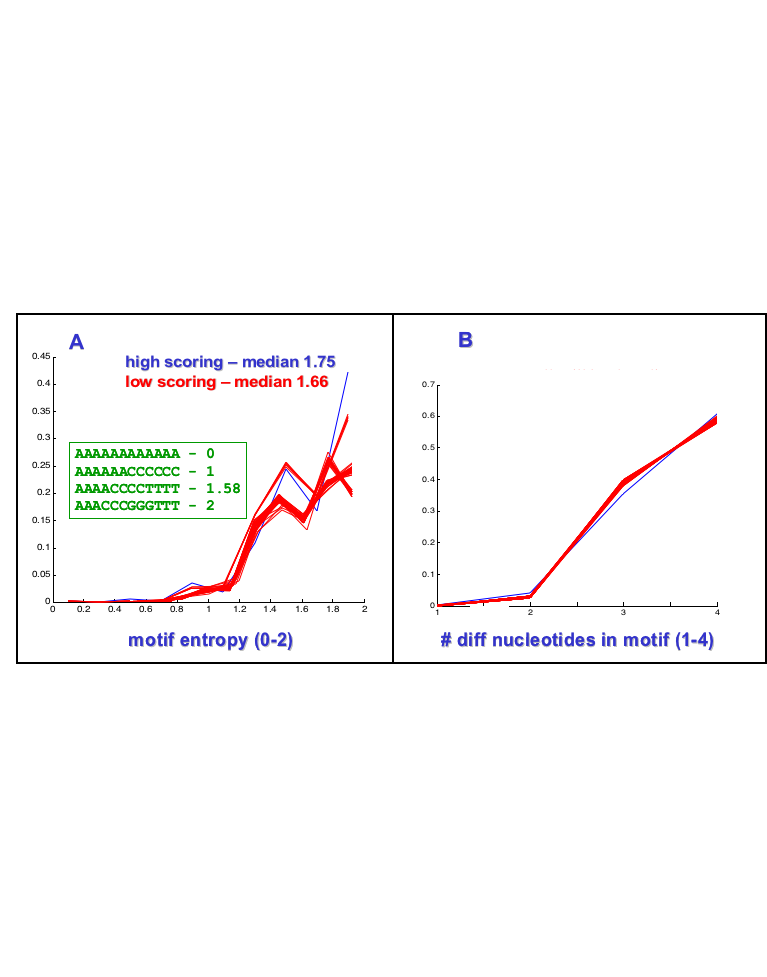


**Figure S3**: A: Distributions of entropy for high scoring k-mers and for 50 random sets of control k-mers. B: distribution of the number of different nucleotides comprising high versus low scoring k-mers (same distributions)
