# Supplementary material for: Functional Characterization of Variations on Regulatory Motifs
Source: PLoS Genet. 2008 Mar 7;4(3):e1000018. doi: 10.1371/journal.pgen.1000018 (PMC2265473; doi:10.1371/journal.pgen.1000018)
Supplement: Figure S4 — Distributions of mean and maximum number of occurrences per promoter for high scoring k-mers versus low scoring k-mers (0.07 MB DOC) [file pgen.1000018.s004.doc]

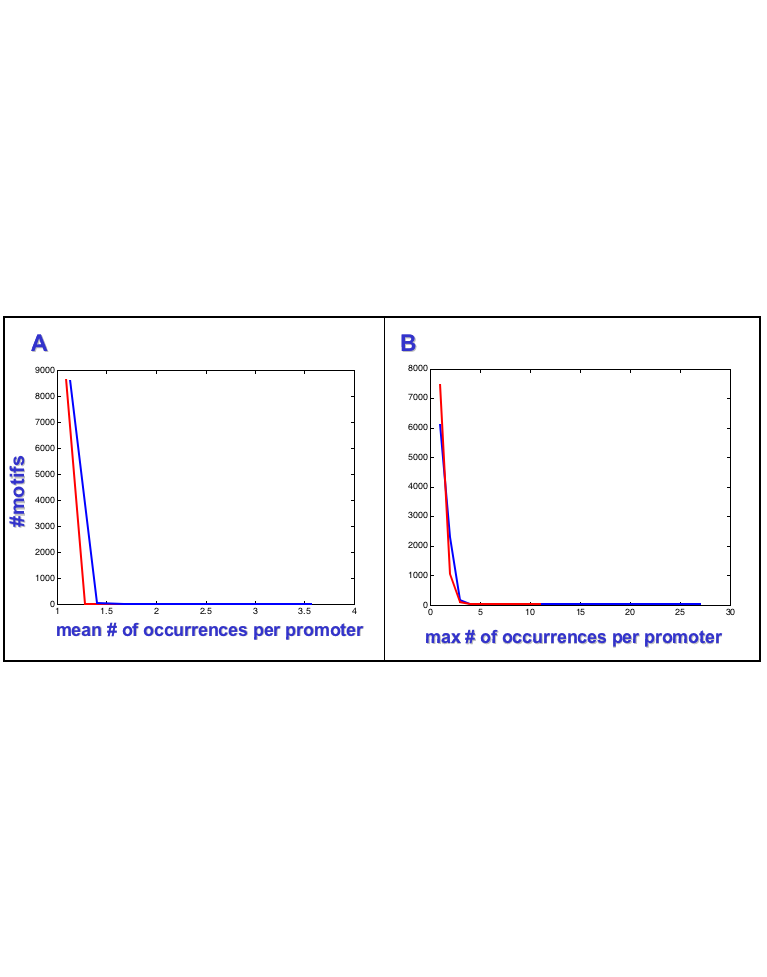


Figure s4: Motif copy number A. The distributions of the mean number of occurrences of each motif per promoter are significantly different (ranksum test - P<10^-300^) for high scoring k-mers (blue) versus low scoring k-mers (red) . B. Distributions of the maximum number of occurrences of each motif per promoter, high scoring (blue) versus low scoring (red) k-mers (ranksum test - P<10^-300^). High scoring motifs tend to appear in a larger copy number.
