# Supplementary material for: Functional Characterization of Variations on Regulatory Motifs
Source: PLoS Genet. 2008 Mar 7;4(3):e1000018. doi: 10.1371/journal.pgen.1000018 (PMC2265473; doi:10.1371/journal.pgen.1000018)
Supplement: Figure S5 — Positional bias of high scoring k-mers. (0.15 MB DOC) [file pgen.1000018.s005.doc]

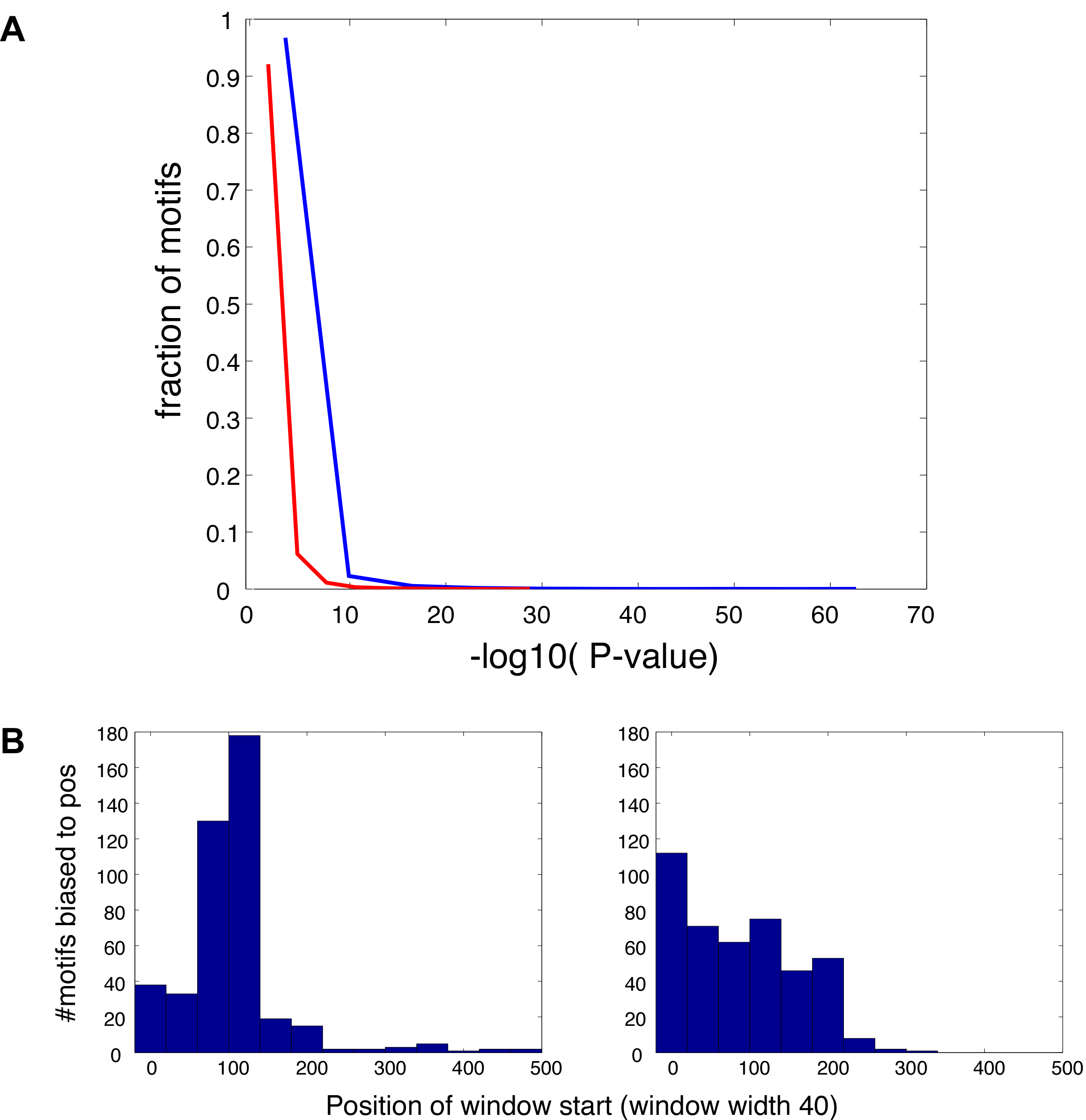


**Figure S5**: Positional bias of high scoring k-mers. A. The distributions of positional bias p-values differ significantly between high (blue) and low (red) scoring k-mers. A greater fraction of the higher scoring k-mers, appear to have a significant positional bias. The best p-value of the high scoring k-mers is 1.08e-066, while the best p-value for the low scoring k-mers is 5.97e-03. B. The preferred positions of positionally biased k-mers (defined as 5% of the k-mers with the most significant p-values) are also significantly different between the two sets of k-mers (ranksum test – P=5.25e-04). Positionally biased high scoring k-mers (left) are primarily located within 80-160 nucleotides from the TSS, whereas the low scoring k-mers (right) can be located throughout the first 240 nucleotides.
