# Supplementary material for: Functional Characterization of Variations on Regulatory Motifs
Source: PLoS Genet. 2008 Mar 7;4(3):e1000018. doi: 10.1371/journal.pgen.1000018 (PMC2265473; doi:10.1371/journal.pgen.1000018)
Supplement: Figure S6 — Plot of normalized EC scores versus evolutionary conservation for the highly scoring S. cerevisiae cell cycle k-mers. (0.09 MB DOC) [file pgen.1000018.s006.doc]

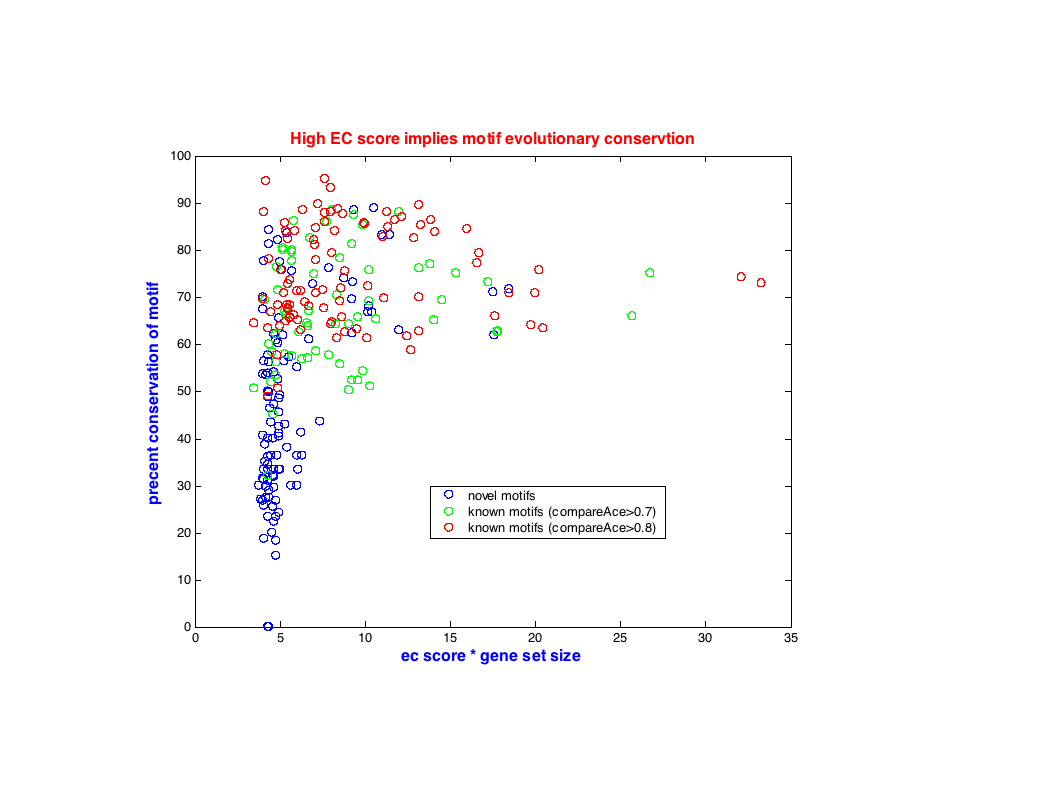
Figure S6: High EC score implies motif evolutionary conservation. Evolutionary conservation in four Saccharomyces species [1] was calculated for the highly scoring S. Cerevisiae cell cycle k-mers (lengths 7-11) and plotted against their normalized EC scores (EC score*gene set size). It is clear from the plot that putative motifs with a higher EC score tend to be more evolutionary conserved. Putative motifs that are similar in sequence to known motifs (compareAce score>0.8) are marked in red, putative motifs for which there is a known motif with a lower sequence similarity (0.7 < compareAce score < 0.8) are marked in green. For these motifs our scoring methodology suggests a refined sequence. Potentially novel motifs are marked in blue

References

1. Cliften P, Sudarsanam P, Desikan A, Fulton L, Fulton B, et al. (2003) Finding functional features in Saccharomyces genomes by phylogenetic footprinting. Science 301: 71-76.
