# Supplementary material for: Functional Characterization of Variations on Regulatory Motifs
Source: PLoS Genet. 2008 Mar 7;4(3):e1000018. doi: 10.1371/journal.pgen.1000018 (PMC2265473; doi:10.1371/journal.pgen.1000018)
Supplement: Figure S7 — Distribution of expression similarities for the different types of single nucleotide substitutions (0.04 MB DOC) [file pgen.1000018.s007.doc]

| A | 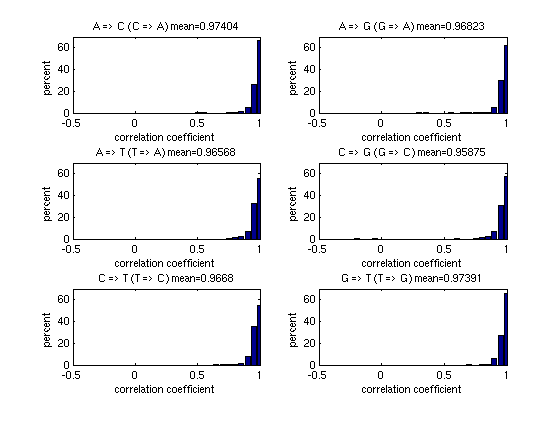 |
| --- | --- |
| B | 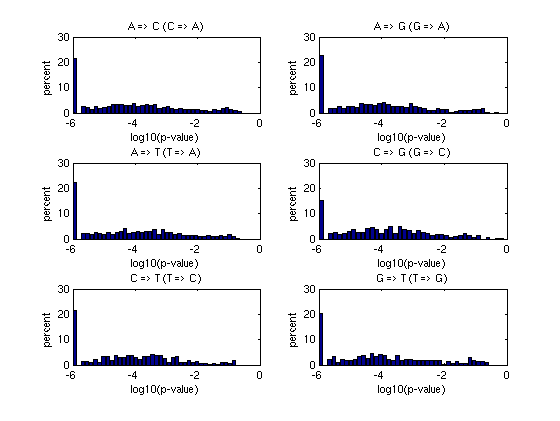 |

**Figure S7 Distribution of expression similarities for the different types of single nucleotide substitutions.**

Pearson correlation coefficients (A) and corresponding p-values (B) were calculated for the mean expression profiles observed in shared conditions of pairs of motifs from the filtered core dataset that differ by a single nucleotide and share at least one condition where they both display significant expression coherence. For the purpose of plotting the p-value histograms very small p-values were conservatively set to 1e-06. Since both the original motif and the substituted motif are members of the filtered core dataset complementary substitutions were pooled together, rather than counting them twice.
