# Supplementary material for: Functional Characterization of Variations on Regulatory Motifs
Source: PLoS Genet. 2008 Mar 7;4(3):e1000018. doi: 10.1371/journal.pgen.1000018 (PMC2265473; doi:10.1371/journal.pgen.1000018)
Supplement: Figure S8 — Comparison of the age of duplication of pairs of paralogs (approximated by Ks) and their tendency to share known regulatory motifs in their promoters (0.12 MB PDF) [file pgen.1000018.s008.pdf]

A

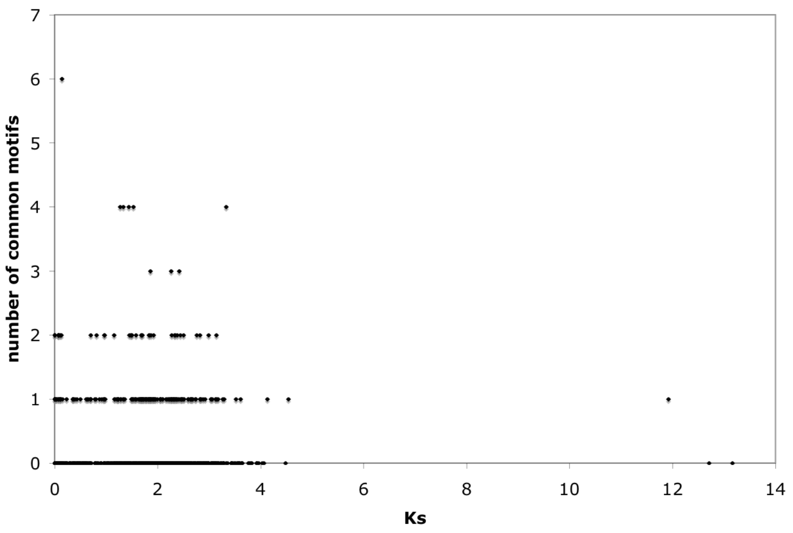


B

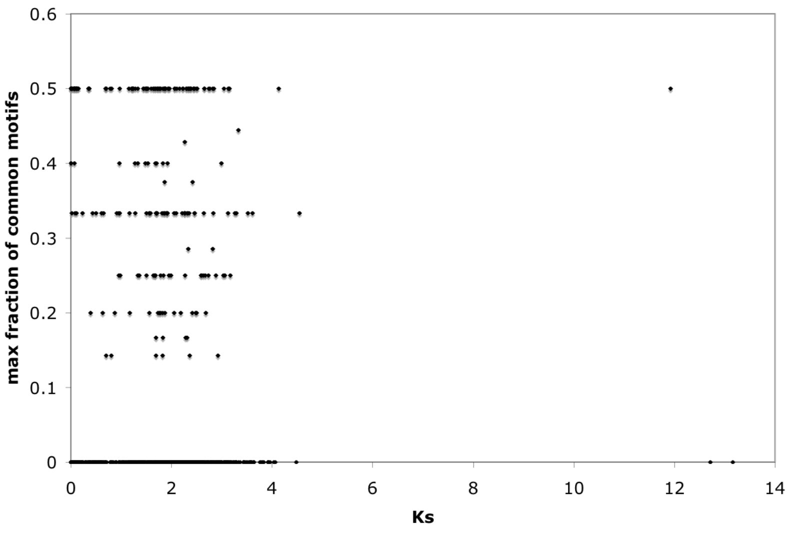


**Figure S8. No relationship between age of duplication and the tendency for common motifs among paralog pairs.** Pairs of paralogs and numbers of known motifs were taken from the work of Kafri et al. [1]. Only pairs of paralogs where a known motif was detected in the promoters of at least one of the paralogs were taken (935 pairs). Ks, the rate of synonymous mutations in the coding region, was used as a proxy for the age of duplication. A. Comparison of age of duplication (Ks) with the number of common motifs in the promoters of the paralogs. B. Comparison of age of duplication (Ks) with the maximal fraction of common motifs, calculated as #common motifs/min(#motifs in paralog1,#motifs in paralog2). If one of the paralogs had no known motifs in its promoter tha maximal fraction of common motifs was set to 0.

**References**

1. Kafri R, Bar-Even A, Pilpel Y (2005) Transcription control reprogramming in genetic backup circuits. Nat Genet 37: 295-299.
