# Supplementary material for: Functional Characterization of Variations on Regulatory Motifs
Source: PLoS Genet. 2008 Mar 7;4(3):e1000018. doi: 10.1371/journal.pgen.1000018 (PMC2265473; doi:10.1371/journal.pgen.1000018)
Supplement: Figure S9 — Distribution of the set sizes of genes examined in constructing the core motif dataset that is relevant for the cell cycle (0.03 MB DOC) [file pgen.1000018.s009.doc]

| 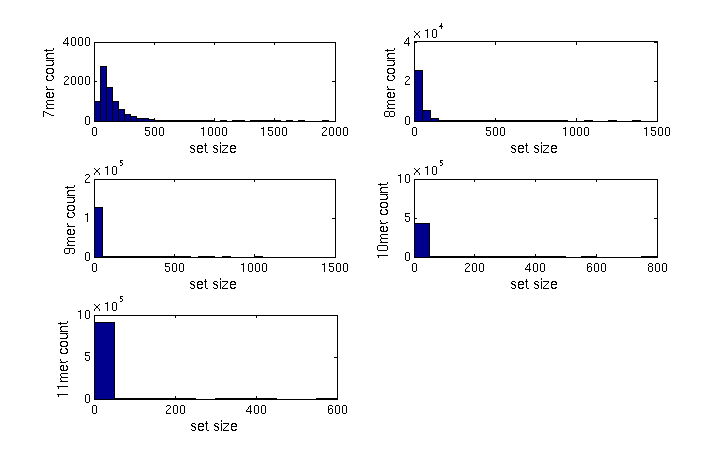 |
| --- |

**Figure S9. Distribution of the set sizes of genes examined in constructing the core motif dataset that is relevant for the cell cycle.** In order to find putative motifs relevant for a certain expression condition we calculated for each k-mer an expression coherence value that is based on the set of genes that harbor it in their promoters and have expression values in the condition considered. The histograms shown were obtained using the expression condition ExpressDB cho cell-cycle.
