# Supplementary material for: Functional Characterization of Variations on Regulatory Motifs
Source: PLoS Genet. 2008 Mar 7;4(3):e1000018. doi: 10.1371/journal.pgen.1000018 (PMC2265473; doi:10.1371/journal.pgen.1000018)
Supplement: Table S2 — Redundancy and uniqueness in the core motif dataset (0.05 MB DOC) [file pgen.1000018.s011.doc]

| motif length | #in data set | # unique | fraction unique | fraction of motifs with derivatives of other length | | | | |
| --- | --- | --- | --- | --- | --- | --- | --- | --- |
|  |  |  |  | 7 | 8 | 9 | 10 | 11 |
| 7 | 1113 | 274 | 0.25 | - | 0.71 | 0.33 | 0.14 | 0.08 |
| 8 | 3472 | 1736 | 0.5 | 0.35 | - | 0.22 | 0.09 | 0.05 |
| 9 | 1859 | 749 | 0.40 | 0.28 | 0.44 | - | 0.23 | 0.13 |
| 10 | 1184 | 322 | 0.27 | 0.23 | 0.35 | 0.43 | - | 0.44 |
| 11 | 982 | 279 | 0.28 | 0.18 | 0.32 | 0.39 | 0.62 | - |
| all | 8610 | 3360 | 0.39 |  | | | | |

**Table S2 – redundancy and uniqueness in the core motif dataset**

For each motif length k we counted the number of unique motifs, i.e. motifs for which there is no derivative of shorter or longer length within the core motif dataset. We also calculated the fraction of motifs of length k that have a derivative of length j, for different values of j such that jk. For our purposes a derivative of a motif is either a subsequence of the motif (in cases we examine shorter lengths than k) or a longer sequence containing the motif (when we examine longer lengths than k).
