# Supplementary material for: Functional Characterization of Variations on Regulatory Motifs
Source: PLoS Genet. 2008 Mar 7;4(3):e1000018. doi: 10.1371/journal.pgen.1000018 (PMC2265473; doi:10.1371/journal.pgen.1000018)
Supplement: Table S3 — Coverage of the Harbison motif set by our core dataset (0.04 MB DOC) [file pgen.1000018.s012.doc]

| Score cutoff | Coverage of our dataset | Coverage of  the Harbison set | Unique Harbison  clusters |
| --- | --- | --- | --- |
| 99 | 1402/8610=16% | 89/102=87% | 72/79=91% |
| 98 | 1528/8610=18% | 93/102=91% | 75/79=95% |
| 97 | 1719/8610=20% | 96/102=94% | 75/79=95% |
| 95 | 2198/8610=25% | 99/102=97% | 77/79=97% |

**Table S3 - Coverage of the Harbison motif set by our core dataset**

A scoring method was devised to assess how likely a given string is to be generated from a given PWM. The score is on a scale of 0 to 100 (see supplementary methods in supporting Text S1). We computed this score for all 8,610 core motifs over the 102 Harbison PWMs. The coverage of Harbison’s motif set was assessed for several different score cutoffs. Note that a single string may match more than one Harbison PWM, because of redundancy in Harbison’s dataset. There are two very long (17 and 18 positions) gapped motif in Harbison’s set, for which we have no match, because our set only covers motifs of length 7-11.
