# Supplementary material for: Functional Characterization of Variations on Regulatory Motifs
Source: PLoS Genet. 2008 Mar 7;4(3):e1000018. doi: 10.1371/journal.pgen.1000018 (PMC2265473; doi:10.1371/journal.pgen.1000018)
Supplement: Text S1 — Validation of the method of construction of the motif dataset and the comparison of this dataset to published datasets. (0.14 MB DOC) [file pgen.1000018.s015.doc]

**Method validation and comparison to published datasets**

**Technical validation**

A potential concern regarding the core motif dataset is that it may be highly redundant in the sense that a long k-mer may be included as shorter lengths that don't include any new information (that is their significance is due to the longer k-mer alone), and *vice versa*. Ideally, if a given motif’s true length is k (i.e. the regulatory protein binds k nucleotides) and we examine the two k-1 long motifs that it contains, or the eight k+1 long motifs that contain it, none of them will obtain as significant a score as the true k-long motif. This is because each of the k-1 long sub-motifs will likely appear in many un-related genes. If on the other hand, we were to examine k+1 long motifs, by extending the original k-mer, then each extension will result in a subset of the original targets, so that although no false positive are added the significance of these motifs will be reduced due to the smaller set size. So taking subsequences of the motif adds noise, whereas examining longer motifs reduces the signal by lowering statistical power, and in both cases sensitivity to the motif, in terms of signal-to-noise ratio, decreases. In short, the length that minimizes the p-value should correspond to the “real” length of the motifs, and shorter and longer versions should score less significantly.

In practice, examination of our core motif dataset reveals that the difference in significance between the “true” motif and derivatives thereof is not always large enough to allow the exclusion of the derivatives from the core motif dataset. The result is a substantial redundancy between motifs generated for different values of k (Table S2). However, reassuringly, for a large fraction of the motifs (~40%) the core dataset contains a single non-redundant instance of the motif with no derivatives (Table S2). This stresses the importance of using various motif lengths in generating the dataset of highly scoring motifs.

**Functional validation**

To validate the ability of our method to identify biologically significant sequence motifs, we tested out whether previously published regulatory motifs score highly using the same scoring scheme. We used the well accepted reference collection of yeast TFBS published by Harbison et al. [1] (hereafter referred to as the Harbison set). Briefly, the Harbison set was obtained by experimentally determining the genomic occupancy of DNA-binding transcriptional regulators under rich medium as well as other growth conditions. Using motif-discovery algorithms the information from genome-wide location data was then combined with phylogenetically conserved sequences and prior knowledge to derive for each regulator its probable specificities [1]. Each motif in the Harbison set is represented by a positional weight matrix (PWM), which specifies for each position in the regulator binding site and each of the four possible nucleotides the likelihood of observing the specific nucleotide at that particular position.

Applying the same FDR cutoff (0.1) to sets of genes containing each of Harbison’s motifs in their promoters, 89/102 (87%) of these gene sets appeared to be coherently expressed in at least one of the examined biological conditions. Sequences generated by these PWMs should have therefore been discovered by our method. The Harbison PWM set is slightly redundant, because it contains several TF families which recognize highly similar binding sites. Therefore, when clustering all of Harbison’s binding sites according to the similarities among their PWM representations, the 102 sites fall into 79 distinct clusters (see supplementary methods below). The Harbison motifs that scored significantly using our method belong to 68/79 (86%) of these clusters, and appear to regulate 39 out of the 40 examined biological conditions. For comparison, only 15/102 (15%) random gene sets, identical in size to the gene sets corresponding to each of Harbison’s motifs, appeared significant in at least one biological conditions (Figure S1). This is only slightly above what would be expected by chance when applying a false discovery rate of 10%.

In the validation step described above, we took the gene sets that correspond to each of Harbison’s TFs and measured their EC scores, without referring to our motif set at all. We additionally assessed our coverage of Harbison’s PWM set by comparing each of our 8,610 core motifs to all of Harbison’s PWMs. We devised a scoring system on a scale of 0-100, which indicates how likely a given k-mer is to be generated from a given PWM (see supplementary methods below). Requiring a match score of 99 we obtained a coverage of 89/102 (87%) of Harbison’s individual PWMs, and of 72/79 (91%) of the non redundant PWM clusters. By relaxing the similarity requirement to 95 the coverage increased to 99/102 (97%) motifs, falling into 77/79(97%) distinct clusters (Table S3). Our coverage of the Harbison set is significantly higher than that of a control set of 8,610 low scoring k-mers (p-value =10e-05).

The overlap between the sets of Harbison TFs that passed each of these validation methods is not complete: 79 Harbison TFs passed both validations, namely their corresponding genes are coherently expressed and there exists a k-mer sequence in our core dataset which matches their binding preferences. Because we applied strict cutoffs in both validation steps (FDR in the first and a requirement for a match score of 99 in the second), each method may have missed some TFs. Together the two validation approaches re-discover 99 out of the 102 Harbison TFs.

For further validation of the regulatory potential of our significant motifs, we examined whether the sets of genes defined by each of the 8,610 core motifs share common biological functions. Such common functionality may indicate a need for common regulation. To assess common functionality, we employed the Functional Coherence (FC) score [2], which uses similarity in Gene Ontology (GO) annotations [3] to quantify the overall functional similarity among a set of genes, in a manner similar to the EC score (see supplementary methods below). A set of genes is functionally coherent if its genes are significantly closer to each other in function than expected by chance given the size of the set. 1,440 (17%) out of the 8,610 motifs that were selected based on significant EC scores, also scored significantly in the functional coherence test, using similarity in GO biological process annotations. For comparison, among 1,000 randomly selected strings from a control set of lowly scoring k-mers (see next section for a full description of the control set), only 3 (0.3%) obtained a significant FC score. This is an additional reassurance that many of our discovered motifs are biologically relevant. FC and EC can be seen as two complementary approaches. A certain overlap is expected between motifs that score highly in EC and those that score highly in FC; Genes that participate in similar biological processes (significant FC) are in many cases (although not always) co-regulated. Genes that are co-regulated (significant EC) are needed in the cell at the same time and thus are likely to belong to the same biological process. However co-expressed genes may also belong to several processes that happen to take place in the cell at the same time.

**Characteristics of significantly scoring k-mers**

We used our set of significantly scoring k-mers to investigate characteristics that may be of relevance to their biological function. For this purpose we compiled a control set of 190,211 low scoring k-mers, that were insignificant in all 40 examined biological conditions, and in addition scored especially low (p-value > 0.8, gene set size>8 ) in at least one of these conditions. We considered various features that may be important for the function of a regulatory motif and for each such feature, defined a quantitative measure, and tested whether it can significantly differentiate between our highly scoring motifs and the control set.

Our significant k-mers were found to have high GC content relative to the yeast AT rich genomic background (Figure S2), to have high entropy (Figure S3), to appear in higher copy numbers than the control k-mers (Figure S4) and to display a preference to distinct positions relative to the transcriptional start site (TSS) in different promoters (positional bias) (Figure S5). Furthermore our significant motifs appeared to be evolutionary conserved in the promoters of four close *Saccharomyces* species (Figure 3 in main text and Figure S6). These are all properties which are known to characterize functional binding sites.

GC content

The nucleotide composition of a motif may be crucial for its function, for instance by allowing it to be readily distinguished from its surrounding promoter sequence. The distribution of normalized GC content (number of GCs/motif length) of the high scoring motifs differs significantly (P<10^-300^) from that of the low scoring k-mers, as seen in Figure S2A. The distribution of the low scoring k-mers peaks close to the mean background promoter GC content (36%), whereas the high scoring motifs have a higher GC content distribution that is comparable to that of the Harbison motif set (ranksum test: P=0.7445). This may allow these motifs to easily detectable by the TF on the background of the AT rich (38% GC, 62%AT) yeast genome.

Entropy

We defined a measure termed motif Entropy to quantify how evenly are the four nucleotides distributed within a candidate motif. That is, we asked whether a functional motif is likely to be composed of an equal amount of all 4 nucleotides or mainly of one or two of the nucleotides. The motif Entropy is defined as follows:

where i can be any of the four nucleotides and q_i_ is the frequency of this nucleotide in the motif. For instance, the sequence ‘AAAAAAAAAAAA’ will have an entropy of 0, ‘AAAAAACCCCCC’ an entropy of 1, ‘AAAACCCCGGG’ an entropy of 1.585 and ‘AAACCCGGGTTT’ an entropy of 2. High scoring motifs that comprise our core dataset, have a significantly (P<10^-22^) higher entropy than low scoring k-mers (Figure S3A), which is expected because they should have a high information content. However the number of nucleotides a motif is composed of is similar for significant motifs and for lowly scoring ones. (Figure S3B). This means that even if a control k-mer is composed of all 4 nucleotides, the distribution is not even, but instead one or perhaps two of the nucleotides is the most prevalent. A simple count of the number of different nucleotides within a k-mer is thus not enough to differentiate between meaningful and nonsense motifs, a more elaborate score such as entropy is needed.

Copy number

Many functional motifs are present in multiple copy numbers in the promoters of the genes they regulate. We thus compared the distribution of motif copy number among high scoring motifs to that of a control set of low scoring ones. The distributions of the maximal number of occurrences of each motif per promoter are significantly different (ranksum test - P<10^-300^). Significant motifs tend to appear in a larger copy number; 29% (2523/8610) of our core motifs appear more than once in a promoter, where the maximal number of occurrences is 27. Only 14% (1171/8610) of the control set k-mers appear more than once in a promoter, where the maximal number of occurrences is 11 (see Figure S4). This is in line with previous reports of TFs that bind multiple binding sites in the same promoter in order to allow stable binding or to permit a graded transcriptional response [1,4] .

Positional bias

A majority of the functional motifs are thought to be located at a preferable distance window from the TSS. This positional bias is most likely needed for their function, and specifically for their cooperation with nearby binding sites. To quantify the positional bias of our motifs, we gathered, for each k-mer, the positions (relative to the TSS) of all its genome-wide promoter instances. These positions were sorted into 40bp wide bins. A positional bias p-value was calculated using a binomial model for the most highly populated interval adapted from Hughes et al. [5]. The distributions of positional bias p-values differ significantly (ranksum test - P<10^-300^) between the significant motif set and the control set (Figure S5A). Although there are k-mers from the control set which display a significant positional bias, their preferred positions differ from those preferred by the high scoring motifs (Figure S5B); The most biased motifs among the high scoring set are located mostly at 80-160 nucleotides upstream of the TSS. The first 80 nucleotides are almost devoid of high scoring motifs, probably because of constraints of the basal transcriptional machinery. This is in line with the findings of Harbison et al., which reported very few binding sites in the region 100 base pairs (bp) upstream of the TSS and a sharp peak in binding site number between 100-200 bp. The most biased motifs among the control set are located anywhere between 0 and 240 nucleotides upstream of the TSS, with the majority at a distance of 0-40.

Evolutionary conservation

Many functional motifs are conserved throughout evolution. Thus, evolutionary conservation is another criterion that may differentiate our high scoring motifs from the presumably non functional low scoring k-mers. Evolutionary conservation across four close *Saccharomyces* species*: S. cerevisiae, S. mikate, S. kudriazevii and S. bayanus* [6] was assessed as follows: for each motif, we counted the percentage of fully conserved (in all aligned species) positions in each of its instances, and averaged over all motif instances. Our motifs showed high evolutionary conservation when compared to a control set of randomized k-mers (in order to preserve the same GC content). In fact the motifs in our set, which scored significantly in the control of progression through cell cycle, were as evolutionary conserved as a set of motifs that were defined solely based on phylogenetic footprinting [7] (Figure 3A in main text). This is striking as conservation was not taken into account in our motif scoring methodology. Moreover it turned out that there is a positive correlation between the normalized EC score (EC score*gene set size ) of a motif and its degree of conservation (Figure S6).

When expanding the conservation analysis to the complete set of high scoring motifs, and not only to those regulating cell cycle, only 17.6% of our motifs had a conservation rate (see supplementary methods below) higher than the 95th percentile of the control set distribution. This may be partly explained by species specific motifs that are likely to be present in every genome. For instance, about 40% of human functional binding sites are estimated to be non functional in rodents [8]. A similar proportion of species specific sites has been observed in yeast [9]. Furthermore we used quite a strict conservation criteria, requiring a position to be maintained in all 4 species in order to be regarded as conserved. The fact that evolutionary conservation appears to be significant in cell cycle motifs, but not in all high scoring motifs, may suggest especially higher conservation across these species in the context of cell cycle regulation.

***Supplementary Methods***

## Comparison of k-mers to PWMs

## A scoring method was devised to assess how likely a given k-mer is to be generated from a given PWM. For each position in the motif and each of the four nucleotides the PWM specifies a weight, which is the base 2 logarithm of the probability that the nucleotide will be observed at this motif position. The score is on a scale of 0 to 100, and is computed by summing up the weights corresponding to the observed nucleotides over all motif positions, and normalizing this score to a scale of 0-100. The scaling is done by subtracting the minimal possible score and dividing by the range of possible scores. For example for the PWM [A: 0.0191 0.0191 0.9733 0.9733 0.0120, C:0.9500 0.9500 0.0074 0.0074 0.0074, G: 0.0117 0.0117 0.0074 0.0074 0.0074 T:0.0191 0.0191 0.0120 0.0120 0.9733] the lowest possible score 0.0455 is obtained for the string GG(C/G)(C/G)(C/G), the highest possible score 4.8198 is obtained for the string CCAAT. After scaling GGCCC will score 0, CCAAT will score 100 and CCATT will score 79.9 ( (3.8585-0.0455)/( 4.8198-0.0455) ).

## Grouping Harbison’s PWM set into distinct clusters

## Highly similar PWMs belonging to the Harbison set were grouped together using hierarchical clustering. The similarity measure used for clustering was the CompareACE score [5]; this method compares two PWMs by aligning them and calculating the Pearson correlation coefficient between the base frequencies of the aligned matrix portions. To prevent spurious matches, CompareACE requires that the aligned portion include at least the six most informative positions in each motif. Using a similarity cutoff of CompareACE score> 0.9, the 102 Harbison PWMs were grouped into 79 distinct clusters.

## The Functional Coherence (FC) score

Functional Coherence (FC) is a term used to describe the extent to which a set of genes is similar in function. Data on biological processes were derived from the GO database [3], which defines a hierarchy of functional annotations. The gene annotations themselves were taken from SGD [10]. The similarity measure between GO functional annotation terms was taken to be the 'semantic similarity', defined by Lord et al. [11]. Given the semantic similarity scores between each pair of GO annotation terms, the similarity score between a pair of genes is defined as follows:

The FC score of a set of genes is defined as the fraction of all 'significantly similar' gene pairs out of all pairs of annotated genes in the set, where significantly similar is determined by a threshold θ:

The threshold θ was set to be the 95th or 90th percentile scores of the distribution of all pairwise similarity scores of the yeast genome. Genes without annotations, or annotated as ‘biological process unknown' were excluded from the analysis. The FC p-value was calculated using random sampling in a similar manner to the EC p-value described in the main methods section.

## Evolutionary conservation rate

Promoter data for four closely related *Saccharomyces* species *S. cerevisiae, S. mikate, S. kudriazevii and S. bayanus* were taken from Cliften et al. [6]. Reference lists of motifs that were defined solely based on phylogenetic footprinting were taken from both Cliften et al. [6] and Kellis et al. [7]. The motif conservation calculation was adapted from Xie et al. [12]. Motif conservation was defined as the fraction of motif positions that are identical across all 4 species. We defined the motif conservation rate separately for each motif as the ratio of conserved motif instances to total occurrences of the motif in the genome. We regarded a motif instance as conserved if it displayed at least 90% conservation. For each motif length (from 7-11), we obtained the distribution of expected conservation rates using a control set of 1,000 random motifs of that length. We took the 95th percentile of the control set distribution as the cutoff defining high conservation and counted the number of motifs with a conservation rate above this cutoff.

***References***
